# Artificial intelligence and stroke imaging

**DOI:** 10.1097/WCO.0000000000001333

**Published:** 2024-11-14

**Authors:** Jane Rondina, Parashkev Nachev

**Affiliations:** High Dimensional Neurology Group, UCL Queen Square Institute of Neurology, University College London, Russell Square House, Bloomsbury, London, UK

**Keywords:** artificial intelligence, generative modelling, stroke imaging

## Abstract

**Purpose of review:**

Though simple in its fundamental mechanism – a critical disruption of local blood supply – stroke is complicated by the intricate nature of the neural substrate, the neurovascular architecture, and their complex interactions in generating its clinical manifestations. This complexity is adequately described by high-resolution imaging with sensitivity not only to parenchymal macrostructure but also microstructure and functional tissue properties, in conjunction with detailed characterization of vascular topology and dynamics. Such descriptive richness mandates models of commensurate complexity only artificial intelligence could plausibly deliver, if we are to achieve the goal of individually precise, personalized care.

**Recent findings:**

Advances in machine vision technology, especially deep learning, are delivering higher fidelity predictive, descriptive, and inferential tools, incorporating increasingly rich imaging information within ever more flexible models. Impact at the clinical front line remains modest, however, owing to the challenges of delivering models robust to the noisy, incomplete, biased, and comparatively small-scale data characteristic of real-world practice.

**Summary:**

The potential benefit of introducing AI to stroke, in imaging and elsewhere, is now unquestionable, but the optimal approach – and the path to real-world application – remain unsettled. Deep generative models offer a compelling solution to current obstacles and are predicted powerfully to catalyse innovation in the field.

## INTRODUCTION

**FIGURE 1:**
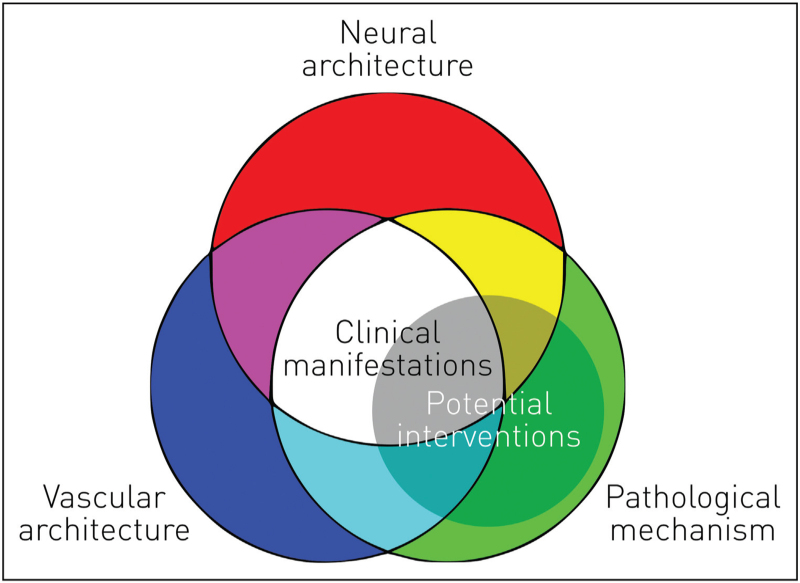
Schematic of the relation between the neural architecture, vascular architecture, and pathological mechanism in giving rise to clinical manifestations and susceptibility to potential interventions.

Artificial intelligence (AI) describes a heterogeneity of analytic methods with one common feature: the combination of great expressivity and flexibility in relating states of affairs of interest – e.g. the anatomy of an ischaemic lesion and its clinical outcome – and minimal prior assumptions on the nature of the relation, placing most of the burden of inference on the data themselves. Though commonly conceived as a means of automating tasks whose complexity currently restricts them to human agents – e.g. obtaining lesion volumes from images of acute stroke – its ultimate role is in enabling the integration of richly described clinical and investigational features to enable accurate decision-making at the individual level (Fig. 1). Such a role implies not approximating human abilities but approaching, more closely than any human being could, the ‘ideal physician’: an agent capable of integrating the widest range of available information – both from the index patient and the wider population – in arriving at an optimally informed decision. The considerable obstacles along the path to this aim – technical, scientific, operational, and cultural – ought not to deflect us from pursuing it, for it is what the fundamental nature of medicine demands. 

**Box 1:**
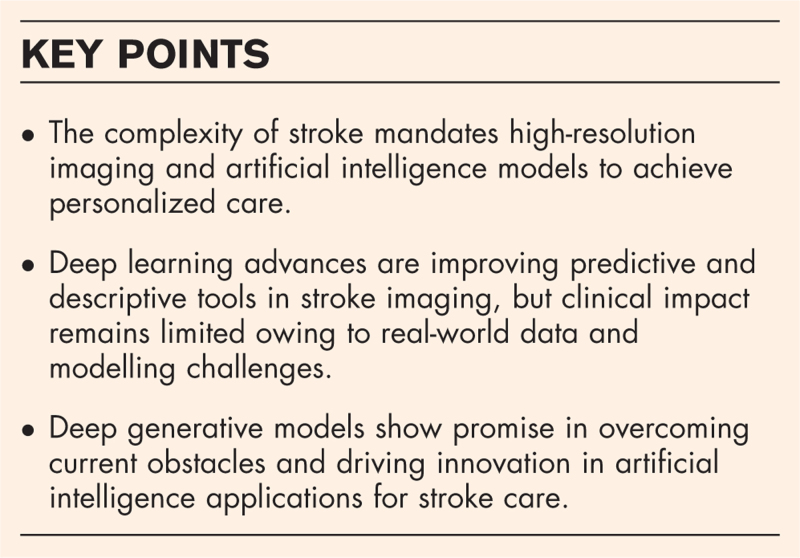
no caption available

Indeed, stroke is an ideal exemplar domain for pioneering such an approach owing to the central role imaging plays in its management and the comparative maturity of AI methods in machine vision [1]. Brain imaging is here established as fundamental to diagnosis and interventional decision-making, and the vast amount of imaging data available, digitally preserved and thereby susceptible to large-scale analysis, alongside the multidisciplinary approaches involved in clinical decision, make stroke one of the potentially pioneering areas for implementation of precision medicine. Specific tools have already been proposed to facilitate diagnosis, and provide information regarding eligibility to interventions to support clinicians in decision making [2,3].

What is perhaps lacking is a general framework for relating component tasks to the overall tasks of approaching the ‘ideal physician’, and consideration of new paradigms in AI, such as deep generative modelling, that could radically change both research and clinical practice.

Note we may distinguish between tasks where the ground truth is currently defined by a human expert – for example, the radiological diagnosis of ischaemic stroke on diffusion-weighted imaging – and where the ground truth is independently determinable – for example, predicting clinical outcomes. In the former case, superiority to a human expert definitionally cannot be established, except where another, more expressive modality is the reference [4,5]. In the latter case, intuitive predictive performance is rarely examined, and the baseline for evaluating the contribution of imaging is typically reductively described based on clinical or manually derived imaging features. It is in this latter class of tasks, where ‘superhuman’ performance is theoretically possible, that the principal value of AI lies.

This paper is structured as follows: We start by discussing contributions in predictive, descriptive, and prescriptive models; we then address inference (with special emphasis on spatial aspects), and contributions of AI to clinical trials. These sections are followed by a discussion of how deep generative models have the potential powerfully to catalyse innovation in each of the areas previously addressed.

## OUTCOME PREDICTION

In the last decade an increasing number of machine learning (ML) applications to predict both risk and poststroke outcomes have been proposed. Despite the substantial body of literature in the area, actual deployment of AI approaches in clinical practice is not yet a reality, as several technical and ethical challenges are involved. However, we seem to be approaching a turning point, as both methodology and regulatory initiatives have advanced rapidly in the last year.

### Prediction of poststroke outcomes

Predicting clinical outcomes based on early acute stroke information, especially as captured by imaging, is imperative to support prognostics, manage resources, address patient expectations, and provide information to clinical trials. In the past year, a variety of different methods have been proposed to predict poststroke outcomes (mainly modified rankin scale). These include the combination of a convolutional neural network (CNN) with a clinical model based on selected features [6], a fusion of an SVM model based on clinical features with a customized CNN based on diffusion-weighted imaging features [7,8^▪^]. Advances in predictive performance – and most importantly its plausible generalizability to real-world clinical situations – are modest. The trend towards using more expressive representations of imaging, such as those made possible by CNNs, remains impeded by the limited scale of available training data, the crudity of standard clinical endpoints, and the abundance of unmodelled factors with a bearing on outcome: problematic aspects that will likely require a different approach, as discussed below.

### Prediction of stroke risk

Given the high incidence of stroke and its devastating impact, the application of methods that can offer insight into prevention has great potential value. Promising initiatives have aimed to predict risk of stroke using AI techniques [9,10,11^▪^,^12^–^14^]. Most studies aiming to predict stroke risk are based on clinical and demographic data alone, inevitably given the limited practicability of surveillance imaging. A noteworthy exception combines clinical data and MRI features to predict risk of stroke in patients with atrial fibrillation [11^▪^]. Stroke recurrence, however, is open to image-based or multimodal modelling, approaches taken by several applications of AI proposed in the past year [15–18] with moderate success.

## DESCRIPTIVE ANALYSIS

A fundamental aspect of any modelling task is the selection of the right descriptive representation of the target system. As the difficulty of high-dimensional predictive modelling shows, the richer the representation, the harder it is to avoid finding a local solution that generalizes poorly (overfitting). Though representation may be wrapped inside the predictive task, the constraints on the scale of available data motivate the pursuit of purely descriptive (i.e. phenotyping) models that can draw widely on data from multiple sources, including outside any task for which outcomes are available. Such phenotyping may also be motivated by mechanistic intelligibility, e.g. the segmentation of the topology of lesions from acute imaging. Applications of descriptive models in stroke imaging involve different aspects, from extracting meaningful features from datasets to identifying patients with specific phenotypes.

### Featurization

The process of transforming raw data into relevant information is known in data science as feature extraction, or featurization. An exemplar task in stroke is the segmentation of lesions from CT or MR imaging, typically relying on a single input modality. Advances in the application of deep learning techniques in segmentation in the last 5 years have led to studies reporting consistently increasing accuracies [19–22]. Evaluations of the rate of progress in achieved fidelity [23–27] suggest we are nearing a plateau, requiring innovations in the general approach, involving image generation [28], multimodal modelling [29,30], and multitask approaches [31]. Validation in deployed healthcare settings will also help determine the level of fidelity real-world contexts require [32].

### Representation learning

Featurization is often conceived as limited to the characterization of individual features, such as the intensity of a voxel on a scan, or the scoring of a cognitive test. But it is properly seen as the broader task of deriving a *latent representation* – potentially from the combination of many ‘native’ features – that is optimized for some downstream task, for example, classification, clustering, or retrieval [33]. Such representations typically encode information projected into a different subspace and can be designed for interpretability, revealing hidden features, or improving performance in another, related task or dataset (transfer learning).

Recent applications of representation learning have been proposed to address challenges in stroke image segmentation. An interesting example is a framework designed to simultaneously perform lesion segmentation and decode time-since-stroke-onset using limited labelled data [34^▪^]. This is achieved by using contrastive learning to detect task-related prior representations from both coarse and fine-grained levels. The former is used to enhance the localization of ischaemic lesions against normal background tissue, whereas the latter is used to reveal semantic relations between the lesioned and healthy regions.

Where, as in imaging, a feature is defined both by its intrinsic signal and its location, representations involve modelling anatomical relationships, i.e. image registration, a task complicated by the difficulty of establishing anatomical correspondences in the setting of anatomy-distorting pathology. One recently proposed solution is a contrastive self-supervised learning framework using a CNN designed for registered images was adapted to work on unregistered images [35]. A pretrained model that classifies Large Vessel Occlusion on registered images was used to guide a model with a different architecture to learn a similar feature representation on unregistered brain CT angiography.

Contrastive learning has played a pivotal role on self-supervised learning in recent years, bringing a major contribution to medical imaging in particular, owing to the difficulty of obtaining labelled images.

### Phenotyping

The manifest clinical heterogeneity of stroke compels a reassessment of established assumptions regarding commonalities of pathophysiology and potential treatment targets. Approaches that help stratify the heterogeneity of traditional stroke subtypes will be key to achieving personalized treatment planning and improving our understanding of the mechanisms of the disease.

Recently, an approach to identify and evaluate new phenotypes of acute noncardioembolic ischaemic stroke based on 92 biomarkers in a multicentre dataset comprising 15 166 patients from 201 hospitals was proposed [36^▪^]. The biomarkers were obtained from clinical, blood, and imaging data and a simplified model was obtained with the most relevant features. Gaussian mixture models were used to obtain clusters. They identified four distinct phenotypes with unique clinical characteristics, possibly unique disease pathophysiology, and significantly different clinical outcomes.

## PRESCRIPTIVE MODELLING

The historical focus on outcome predictive models is at odds with the primary objective of medicine: predicting outcomes conditioned on treatment, i.e. *prescriptive* models. It is assumed that prescriptive guidance can only be obtained from randomized controlled trial (RCT) data, typically involving descriptions of each patient simple enough for treatment effects to be estimated with conventional statistics. But the manifest heterogeneity of stroke implies population-average treatment effects may be a poor guide to individual susceptibility. Randomization offers no protection here, for the error inheres in the absence of homogeneous treatment susceptibility RCTs fundamentally assume. A general platform for modelling individualized treatment effects sensitive to lesion topology has recently been proposed to address this problem [37^▪^]. It enables simulation of heterogeneous treatment effect sizes and variability, allowing explicit modelling of response failure and spontaneous recovery in evaluating strategies for inferring individualized treatment effects, even in the context of nonrandom treatment allocation characteristic of observational data. This challenging problem requires joint innovation in predictive and representational modelling so at to render causal models tractable in the setting of rich data, such as neuroimaging.

## SPATIAL INFERENCE

Any outcome model – prescriptive or predictive – must implicitly capture the functional organization of the neural substrate focal lesions disrupts to produce a clinical deficit. Since most of the data on which functional brain mapping rests is correlative, derived from functional signals that merely accompany behaviour and need not be necessary for it, it falls on stroke as the commonest cause of focal brain injury to inform our understanding of the functional architecture of the brain with substantive causal power. This motivates the development of lesion-deficit models that explicitly reveal the underlying neural dependence from paired lesion-deficit data. Though commonly approached with mass univariate models analogously to the analysis of functional imaging, under the assumption of a simple co-occurrence structure of lesions, this task requires complex multivariate models sensitive to the wide variety of lesion morphologies, whose optimal architecture remains a matter of debate [38,39]. Nonetheless, in the past year, spatial inference studies in stroke have addressed some specific tasks, such as mapping NIHSS structure [40], and learning physics-informed spatio-temporal properties of CT perfusion images [41].

## CLINICAL TRIALS

The challenges of treatment heterogeneity at the point of prescription aside, the development and evaluation of new drugs requires radical innovation if the striking inefficiency of interventional research and development is to be substantially improved [42]. Although AI is already seeing applications in the early stages of drug discovery, principally in the search for novel targets, there is increasing recognition of a wider scope for benefit. In trial design, tasks related to dosage of drugs, number of patients, and what data to collect are increasingly being facilitated by AI methods. Examples include algorithms to predict whether a trial will succeed, to obtain safety and efficacy information from kindred other clinical trials, extract data from unstructured reports, curate, harmonize and complete data, and help identify subgroups with heterogeneous treatment responses as outlined above.

## THE RISE OF DEEP GENERATIVE MODELS

There is a striking disconnect between the potential power of AI to extract high-fidelity actionable signals from rich data and success in real-world clinical translation. The root cause is the difficulty of building models that are both sufficiently expressive to capture individual variability and robust to the noise, bias, incompleteness, and distributional variability of real clinical data, especially in the setting of limited labelled cases. Overcoming this difficulty requires not merely better *discriminative* models—i.e. those that identify boundaries critical to some decision, such as outcome prediction – but *generative* models that attempt to learn the data-generating process itself. Such models allow intelligence to be drawn from multiple sources, enabling efficient transfer learning across dissimilar datasets, and illumination of the multiple factors – biological, pathological, and instrumental – that shape the data, and must be accounted for *prior* to looking for any decision boundary within it. In brain imaging, generative models are being proposed to address multiple tasks such as co-registration, super-resolution, enhancement, missing data imputation, multimodal segmentation, cross-modality image synthesis, brain network analysis, fMRI encoding and decoding [43]. Such models ideally integrate information across multiple modalities, but even applying them solely to 3D data such as brain imaging demands both substantial data scales and very high performance computing, as well as algorithmic innovation [44].

Success, however, has the potential powerfully to facilitate all downstream tasks. In prediction, for example, they can help address imbalance and missing data. A recent example is the application of GANs to augment a dataset of patient records and demographics [45], where classifiers were trained on the original and augmented data and obtained enhanced predictive accuracy across multiple models despite the dataset imbalance. In feature extraction, generative models can be used to define a baseline against which a lesion arises as an anomaly with minimal or no training. An example is the application of GANs to segment stroke lesions on follow up noncontrasted CT scans [46], where haemorrhagic and ischaemic stroke lesions removed from follow-up noncontrasted CT scans by generating difference maps with a lesion and baseline noncontrasted scans without a lesion. In data conditioning – enhancing a dataset so as to make it more useful – generative models can be used to synthesize imaging modalities from noise or incomplete data. An example is the experimental verification of image reconstruction using GANs in a new modality of images (CCEIT – capacitively coupled electrical impedance tomography) of brain stroke [47]. This is a new imaging modality that has not yet been introduced into clinical practice, so numerical simulation is the only possible way to acquire training data. The authors suggest that CCEIT measurements combined with generative reconstruction can potentially obtain conductivity spatial distribution imaging in the human head with differences in dielectric properties corresponding to changes observed in brains of stroke patients.

Generative models also offer the possibility of simulating interventions, making this approach a powerful resource for prescriptive models. A recent application [48] proposed a VAE model based on both imaging and tabular data to predict treatment outcome. The model was evaluated on a clinical dataset of intracerebral haemorrhage with improvement in treatment outcome prediction in comparison to other treatment effect estimation techniques. Another recent study [49] proposed a new pretraining and fine-tuning framework to estimate the causal effect of a treatment. Unlabelled data were extracted from real-world medical datasets and encoded as sequential input for pretraining using a transformer-based model with an unsupervised learning objective to generate patient representations. Downstream datasets with labelled treatment and outcome were created for treatment effect estimation using established randomized clinical trials. Comparative effectiveness of two treatment effects in reducing the risk of stroke for patients with coronary artery disease were evaluated.

Regarding topological inference in stroke, generative models can address the challenge of lesion-deficit relationships. An innovative approach [50] proposed a Bayesian deep generative modelling of volumetric data based on a variational auto-encoder that learns the joint distribution of lesions and deficit labels in terms of an anatomical latent neural substrate.

Clinical trials can also benefit from generative models. One of the potential applications is the reduction in the number of patients needed for a trial with resources as ‘digital twins’, used to predict how the same patient would have progressed in the control group and compare outcomes. This approach can reduce the need for ethically questionable placebo arms [42].

## CONCLUSION

AI has great potential to transform both research and stroke care through better predictive, descriptive, prescriptive and inferential models than are currently possible, while contributing to making the best possible use of imaging resources. However, the integration of AI into clinical practice requires careful consideration of technical, regulatory, and practical challenges to maximize its utility. Deep generative models offer currently the most plausible path to solving these challenges, and invite discussion of the sophisticated data, computational, regulatory, and cultural changes their development will require if the potential benefits to patients and society in general are to be realized.

## Acknowledgements

*None.*

### Financial support and sponsorship

*Wellcome Trust and the UCLH NIHR Biomedical Research Centre.*

### Conflicts of interest

*P.N. is a director of Hologen, a healthcare AI company.*

## REFERENCES AND RECOMMENDED READING

Papers of particular interest, published within the annual period of review, have been highlighted as:

▪ of special interest
▪▪ of outstanding interest
